# The Provision for Cases of Senile Insanity

**Published:** 1915-05-22

**Authors:** 


### The Hospital

The Workers' Newspaper of Administrative Medicine and Institutional Life, Administration, National Insurance and Health.

No. 1509, Vol. LVIII. SATURDAY, MAY 22, 1915. PRICE ONE PENNY

### THE PROVISION FOR CASES OF SENILE INSANITY.

At a recent meeting of the Romford Board of Guardians attention was drawn to the number of aged inmates of the workhouse who had been transferred to the County Asylum during the last twelve months. It was proposed to appoint a committee to consider the matter with a view to treating more of such cases in the workhouse. The matter is one to which attention has frequently been drawn by the Board of Control in their annual reports, and which has often formed the subject of mutual recriminations by Boards of Guardians and Asylum Committees. The root of the difficulty is to be found in the fact that whilst the Lunacy Acts have thrown upon the County and certain Borough Councils the duty of providing institutions for the insane, one section of these Acts empowers the medical officer of the workhouse to detain there any mental patient who in his opinion can be properly treated within its walls. The result is that the problem of insanity is being dealt with by two authorities, neither of which is fully cognisant of the work and difficulties of the other. The Councils have devoted themselves mainly to the provision of accommodation for acute and dangerous types of insanity and have assumed that the Guardians would make provision in their workhouses for the quiet, incurable, and demented class and for cases of senile insanity. Few of them have erected the simple buildings which would be adequate for the treatment of these incurable cases, and they naturally object to seeing the costly institutions they have provided filled with cases which can derive no benefit from them. On the other hand, few Boards of Guardians have made any satisfactory provision in their workhouses for the separate treatment of the cases of insanity they are authorised to retain. Even where separate wards have been provided the staffing is usually inadequate, and far too much reliance is placed upon pauper help.

In London the problem has been further complicated by the creation of the Metropolitan Asylums Board. Amongst the duties allotted to this Board at its formation were the reception and relief of such insane paupers of the chronic or imbecile class as could be lawfully retained in a workhouse. The Board has interpreted this clause in practice as applying mainly to the imbecile class, and has made full and elaborate provision for its reception and treatment. The special requirements of the senile insane have been overlooked, and the Board objects as strongly as the London County Council to admitting them to their institutions. When the Royal Commission on the Care and Control of the Feeble-Minded was sitting, Mr. Helby, who was then Chairman of the Board, in giving evidence, said, "Boards of Guardians appear to send all the senile decay people to us, certifying them as imbeciles—a most cruel thing. Our Tooting Bec Asylum is practically nearly full of what are termed imbeciles of that character, nothing more or less than old men and women suffering from senile decay. In my opinion the proper place for this class of patient would be an infirm ward in the workhouse infirmary, where the relatives could visit them."

Mr. Helby's pronouncement is a very inaccurate statement of the problem. Many of these cases of senile insanity are amongst the most troublesome to deal with, and need all the care that can be given by skill and experience. Their noisy and restless behaviour, especially at night, and their unclean and offensive habits render them a source of distress to others and make it impossible to retain them in the ordinary wards of a workhouse or infirmary. In 1905 the Lunacy Commissioners addressed a series of questions to the mental hospital medical superintendents on the suitability for retention in workhouses of the senile cases sent to them. The replies received showed that the majority of the cases sent were unsuitable for workhouse care. Certain of the superintendents drew attention to the lack of proper accommodation and supervision in workhouses. Dr. Jones, of Claybury, said that all the cases sent there could only be fitly treated in a mental hospital with a large and competent staff. On the other hand, Dr. Stewart, of the Leicester and Rutland Asylum, thought that none of the cases he had received need have been sent had there been proper provision made for their care in the workhouses. Here we come to the crux of the whole matter. The Asylums Committees do not want these cases and the Guardians have not made proper provision for them. Each authority thinks it is the duty of the other to make the necessary arrangements. This unsatisfactory state of affairs is another example of the evils produced by having two authorities dealing with the same kind of work. It is an additional argument in favour of placing all public provision for the treatment of the sick in any locality in the hands of one body. If the County Councils were made responsible for all those whose mental or bodily infirmity is dealt with out of public funds the problem could be solved at once. The one authority would have the whole facts before it, and would be in a position to adopt a definite system of classification and to arrange the accommodation at its disposal in such a way as to ensure the best provision being made.

